# Poetry in motion: catching molecules in action

**DOI:** 10.1107/S2052252524002082

**Published:** 2024-03-05

**Authors:** Edward N. Baker

**Affiliations:** a School of Biological Sciences, University of Auckland, Auckland, New Zealand

**Keywords:** protein dynamics, time-resolved crystallography, serial femtosecond crystallography, Laue diffraction, editorial

## Abstract

Researchers have long sought to ‘see’ proteins and other macromolecules in motion, to better understand their functions. Technological developments, notably advances in serial crystallography, are now making these dreams a reality, heralding a new era of kinetic crystallography.

Proteins and the complexes they form are marvels of nature, beautiful in their artistic elegance, while at the same time essential as the ‘working molecules’ of life. Currently, some 216 000 experimentally determined protein 3D structures are archived in the Worldwide Protein Data Bank (wwPDB). When the number of protein structures modelled by predictive algorithms such as those of *AlphaFold2* are added in, we have more than 200 million to consider.

What do all these proteins do? And how do they perform their functions? Do their structures change? Even in the very first protein crystal structure to be determined, that of myoglobin, it became evident that flexibility and dynamics must be important, when the question of how oxygen could access the buried iron atom was considered. Direct evidence of structural motions was hard to come by, however. Flexible regions in a protein could be inferred from high *B* factors or absence of electron density, and structures determined in different crystal environments sometimes revealed changes in conformation. These could be quite large, for example domain movements, and are often correlated with functional properties. Solution scattering methods such as small-angle and wide-angle X-ray or neutron scattering help confirm that these are innate molecular properties, while high-field solution NMR, developed in the 1980s, likewise pointed to flexible regions in protein molecules.

The goal of structural biology, however, is not just to picture biological macromolecules, but to understand function. Ideally, one would like to follow the complete reaction cycles of proteins throughout their course, in as much detail as possible. Steps towards this goal have sometimes been possible by trapping a protein at different points in its reaction cycle, for example by determining crystal structures in the presence of relevant substrates, inhibitors, products, cofactors, or other molecules or ions, thus building up a series of snapshots of a protein in action. To go further, however, requires the crystallographic experiments to be conducted at time scales approaching those of the reactions involved, milliseconds or less.

The point of this editorial is to emphasize the view that we are on the cusp of a golden age for kinetic crystallography. The groundwork has been laid over the past 40 years, inspired first by the pioneering work by Keith Moffat and John Helliwell, whose development of Laue diffraction gave the first time-resolved views of proteins in action (Moffat, 2019 ▸). Those experiments made use of very short pulses of polychromatic radiation but were limited in the kinds of reactions that could be followed and by the light sources then available. The advent of free-electron lasers, providing femtosecond X-ray pulses, led to the suggestion (Neutze *et al.*, 2000 ▸) that these could offer the possibility of a new era of time-resolved X-ray studies. This has now been realized in the new science of serial femtosecond crystallography (SFX) (Schlichting, 2015 ▸), and has recently expanded further as fourth-generation synchrotron facilities come online. These offer very high-intensity and coherent X-ray beams which, coupled with advances in detector technology, bring serial X-ray crystallography into wider reach. In parallel, similar developments in electron crystallography have demonstrated the potential for serial ED to be carried out on a scanning transmission electron microscope, for applications both in structural biology (Bücker *et al.*, 2020 ▸) and materials science (Hogan-Lamarre *et al.*, 2024 ▸). To this expanding repertoire of diffraction methods can be added the exciting advances in cryo-EM which bring even the largest multiprotein complexes and biological machines into focus. These enable multiple conformations to be visualized, when present, and further methods development is expected.

With such a rapidly expanding toolkit available (Muench *et al.*, 2019 ▸), what challenges remain? There are still key decisions to be made prior to any data collection: choice of a reaction with an amenable timescale; how to obtain a sample appropriate for the method of choice; how to initiate a reaction so that essentially all the molecules are synchronized; whether to use room temperature or cryo-trapping methods; and whether spectroscopic tools can be used to follow the reactions in parallel. Already a lot of options are available (Caramello & Royant, 2024 ▸) and fascinating accounts of successful time-resolved analyses of enzymatic reactions are now appearing (*e.g.* Wilamowski *et al.*, 2022 ▸).

Today I am just an admiring observer, and it is inevitable that many researchers will find research of this kind out of reach, dependent as it is on access to major facilities. But these are issues that can be resolved (Argyriou, 2024 ▸) and in the meantime we can all take pride in the exciting ways in which ‘our’ science is broaching new frontiers.
